# The Reliability of Pelvic Floor Muscle Bioelectrical Activity (sEMG) Assessment Using a Multi-Activity Measurement Protocol in Young Women

**DOI:** 10.3390/ijerph18020765

**Published:** 2021-01-18

**Authors:** Łukasz Oleksy, Anna Mika, Iwona Sulowska-Daszyk, Ewelina Rosłoniec, Renata Kielnar, Artur Stolarczyk

**Affiliations:** 1 Orthopaedic and Rehabilitation Department, Medical University of Warsaw, 02-091 Warsaw, Poland; loleksy@oleksy-fizjoterapia.pl (Ł.O.); drstolarczyk@gmail.com (A.S.); 2 Oleksy Medical & Sports Sciences, 37-100 Łańcut, Poland; 3 Institute of Clinical Rehabilitation, University of Physical Education in Kraków, Al. Jana Pawła II 78, 31-571 Krakow, Poland; iwona.sulowska@awf.krakow.pl; 4 Department of Physiotherapy, Poznań University of Physical Education, 66-400 Gorzów Wielkopolski, Poland; ewelinarosloniec@gmail.com; 5 Institute of Health Sciences, Medical College of Rzeszów University, 35-315 Rzeszów, Poland; kielnarrenata@o2.pl

**Keywords:** surface electromyography (sEMG), pelvic floor muscles, Glazer protocol, multi-activity sEMG protocol, reliability

## Abstract

The aim of the study was to determine the between-trial and between-day reliability of the Glazer protocol and our multi-activity surface electromyography (sEMG) measurement protocol for pelvic floor muscle (PFM) evaluation. The bioelectrical activity of PFM was collected using an endovaginal electrode in 30 young, Caucasian, nulliparous women (age 22–27, 168.6 ± 5.1 cm, 57.1 ± 11.8 kg). The between-trial and between-day reliability of the original Glazer protocol and the new multi-activity sEMG protocol were assessed during the following phases: pre-baseline rest, phasic (flick) contractions, tonic contractions, endurance contraction, and post-baseline rest. The Glazer protocol was characterized by poor and moderate measurement reliability. The time-domain parameters for the rise and fall of the signal amplitude and median frequency showed poor between-trial and between-day reliability. The mean and peak amplitudes indicated mainly good between-trial and moderate between-days reliability. Our protocol showed moderate to excellent reliability of both time-domain and quantitative parameters of muscle recruitment. In our protocol, the frequency-domain parameters describing muscle fatigue demonstrated much higher reliability than in the case of the Glazer protocol. The most important information obtained in this study was the significant improvement of diagnostic validity in PFM bioelectrical activity evaluation. The higher reliability of our sEMG protocol compared to original Glazer protocol allowed us to suggest that protocol modifications and changes in sEMG signal processing methods were effective in the improvement of PFM assessment quality. The new parameters calculated from the sEMG signal proposed in our sEMG protocol allowed us to obtain additional clinically important information about PFM dysfunctions regarding specific deficits of muscle contraction such as decrease in muscle strength; endurance or coordination related to, e.g., stress urinary incontinence; or pelvic floor muscle imbalance after childbirth.

## 1. Introduction

The pelvic floor muscles (PFM) have dual function—providing trunk stability and continence [1,2,3]. It has been reported that women with urinary incontinence have weakened PFM strength and endurance compared to women without pelvic floor dysfunctions [4,5,6,7]. Because PFM dysfunctions are very common, there is a need to create a valid and effective diagnostic method. Many authors have described the use of intrapelvic surface electromyography (sEMG) as an easy and non-invasive method for PFM evaluation [8,9,10]. This method allows assessment of the level of muscle fatigue, as well as chronic overload or disturbed coordination [11,12,13,14]. The bioelectrical activity of the PFMs is commonly evaluated with the Glazer protocol [15,16,17]. The intrapelvic sEMG assessment in the Glazer protocol includes the following series of muscle contractions and relaxations: pre-baseline rest, phasic contractions, tonic contractions, isometric contraction for muscle endurance evaluation, and post-baseline rest. The sEMG signal analysis includes average sEMG amplitude, recruitment and recovery latencies, changes in spectral frequency, and sEMG amplitude variability [16]. Nonetheless, the Glazer protocol is based on a non-normalized sEMG signal, which due to signal variability is its main weakness [16,18].

Some authors have assessed the reliability of PFM bioelectrical activity, but the vast majority took only selected types of muscle contraction into account, such as short maximal contraction or endurance contraction lasting a several dozen seconds [6,7,8,14,18,19]. Therefore, in these works, the real measurement reliability of these tests was not shown, being restricted to only its selected parts. Moreover, Glazer et al. [18] did not evaluate their whole sEMG protocol for reliability, but only one of its phases—a 10-s contraction.

In the literature, the reliability of PFM sEMG measurements are equivocal. In some studies, good reliability has been reported regarding the sEMG signal evaluated both qualitatively and quantitatively [6,7,8,9,14,18,19]. However, as underlined by Auchincloss et al. [20], in each of these studies, different vaginal probes were used and different measurement protocols were applied, making cross-study comparisons impossible. Between-session reliability of sEMG recorded using intravaginal probes was variable, and much better between-trial than between-day reliability was suggested [18]. Auchincloss et al. [20] have also reported that between-trial reliability was acceptable to record PFM sEMG, but the test–retest reliability between days was poor. However, in the Glazer et al. study [18], between-day reliability of the sEMG data was strong and significant, but the authors reported a correlation (*r* = 0.86, *p* < 0.001) of sEMG amplitude only between two measurements. Therefore, due to the weak statistical methods employed, these findings regarding reliability should be considered with caution.

Most often in subjects suffering from incontinence, we can observe complex changes in PFM neuromuscular control, strength, endurance, and coordination. Therefore, for proper diagnosis, a complex evaluative approach is required [3,10,15,21]. In the majority of the studies, the results of PFM assessment with sEMG have been reported only on the basis of PFM activity during contraction and relaxation state [4,14,18,19,22]. Some authors performed assessment during 30 s of contraction evaluating changes in PFM fatigue [23]. However, because the changes in PFM are multifactorial, the clinical evaluation should include different types of muscle contractions, which are similar to those performed during daily life [10,21,24].

When sEMG is used clinically as well as in research, the reliability of this measurement is fundamental for interpretation of the obtained results. Despite the common use of sEMG in PFM function evaluation, the reliability of this measurement has not been fully established. Therefore, there is a need to verify the reliability of PFM sEMG evaluation including different types, strength, and duration of muscle contractions, and also to reduce the sEMG signal variability by appropriate signal processing.

Because there is a lack of studies in which the reliability of PFM sEMG assessment would be comprehensively evaluated, in this study, the aforementioned issue is undertaken for the first time. The purpose of this study is to determine the between-trial and between-day reliability of the multi-activity sEMG measurement protocol for PFM clinical evaluation, which includes all phases described by Glazer et al. [18]. Moreover, because the original Glazer protocol has weaknesses related to sEMG signal variability, making its clinical validity vulnerable to errors, we proposed additional parameters obtained during sEMG signal processing, which may increase the quality of the sEMG signal and, therefore, improve the reliability of PFM assessment. This is the first study in which the reliability of PFM bioelectrical activity is comprehensively evaluated with the broad aspect of muscle contraction and full signal processing standardization. Moreover, in this study, the reliability of full Glazer protocol was evaluated for the first time.

## 2. Materials and Methods

### 2.1. Participants

In this study, 30 young, Caucasian, nulliparous women (age 22–27, 168.6 ± 5.1 cm, 57.1 ± 11.8 kg) without pelvic floor muscle ailments were evaluated. They were recreationally active and did not engage in regular physical training. They did not have any symptoms of urinary incontinence and did not experience any spinal pain in the 6-month period prior to enrolment in the study. They were informed in detail about the research protocol and gave their written informed consent to participate in the study. All procedures were performed in accordance with the 1964 Helsinki declaration and its later amendments. Approval of the Ethical Committee of Rzeszów University (4 January 2015) was obtained for this study.

### 2.2. Procedures

#### The sEMG Measurement

Bioelectrical activity of the PFM was collected using the Life-Care two-sided endovaginal electrode (Everyway Medical Instruments Co., Ltd., Taiwan). The signal was registered with 16-bit accuracy at a sampling rate of 1500 Hz using the Noraxon G2 TeleMyo 2400 unit (Noraxon USA, Inc., Scottsdale, AZ, USA) [12,25].

PFM activity was recorded in supine position with a pillow underneath the participant’s head. The hips and knees were gently flexed, supported by a pillow under the knees, and the lumbar spine was in a neutral position. The women were asked to empty their bladder prior to electrode application. After electrode application, to better familiarize the subjects with testing procedures, we got them to perform a short trial of phasic, tonic, and endurance contractions. After 10 min of rest in supine position, the measurement was performed. During all measurements, participants were verbally instructed to perform the PFM contraction without the use of the abdominal, gluteal, or hip adductor muscles.

Visit 1—For the between-trial reliability, 2 measurements were performed with 15 min of rest between them. The reliability was calculated between the first and second measurement.

Visit 2—For the between-day reliability, 1 measurement was performed 2–3 days later, during the second visit in the laboratory. The reliability was calculated between the first measurement from Visit 1 and the measurement from Visit 2.

The between-trial and between-day reliability of the original Glazer protocol and the new multi-activity sEMG protocol were performed during a single measurement. The measurement of PFM sEMG activity included all phases required by both protocols:

1. One 60-s rest (pre-baseline).
2. Five phasic (flick) contractions (2-s contraction with a 2-s rest in-between).
3. Five phasic (flick) contractions (2-s contraction with a 10-s rest in-between).
4. Five 10-s tonic contractions, with a 10-s rest in-between.
5. One 60-s endurance contraction.
6. One 60-s rest (post-baseline).

The Glazer protocol consisted of 5 activities [17]:

1. One 60-s rest (pre-baseline)—the women were instructed to feel the PFM in rest.
2. Five phasic (flick) contractions—the women were instructed to contract the PFM as quickly as possible (2-s contraction with a 2-s rest in-between).
3. Five 10-s tonic contractions, with a 10-s rest in-between—the women were instructed to contract the PFM as strongly as possible, hold the contraction for 10 s, and then fully relax the PFM after contraction remaining relaxed for 10 s.
4. One 60-s endurance contraction—the women were instructed to contract the PFM at such a level as to hold it for 60 s.
5. One 60-s rest (post-baseline)—the women were instructed to feel the PFM in rest.

The following sEMG signal parameters were calculated for the Glazer protocol [17]:

1. One 60-s rest (pre-baseline):
  Average mean amplitude (μV)
  Mean amplitude variability (%)
2. Five 2-s phasic (flick) contractions with 2-s rest in-between:
  Average peak amplitude (μV)—the result was the mean value from 5 contractions.
  Time before peak (s)—the result was the mean value from 5 contractions.
  Time after peak (s)—the result was the mean value from 5 contractions.
3. Five 10-s tonic contractions, with a 10-s rest in-between:
  Average mean amplitude (μV)—the result was the mean value from 5 contractions.
  Average peak amplitude (μV)—the result was the mean value from 5 contractions.
  Time before peak (s)—the result was the mean value from 5 contractions.
  Time after peak (s)—the result was the mean value from 5 contractions.
4. One 60-s endurance contraction:
  Median frequency (Hz)
  Average mean amplitude (μV)
  Mean amplitude variability (%)
5. One 60-s rest (post-baseline):
  Average mean amplitude (μV)
  Mean amplitude variability (%)

Our multi-activity sEMG protocol consisted of 5 activities:

1. One 60-s rest (pre-baseline—divided into 3 intervals: I-5s, II-5s, III-50s)—the women were instructed to feel the PFM in rest.
2. Five 2-s phasic (flick) contractions, with a 10-s rest in-between—the women were instructed to contract the PFM as quickly as possible, and then quickly and fully relax the PFM immediately after contraction.
3. Five 10-s tonic contractions, with a 10-s rest in-between—the women were instructed to contract the PFM as strongly as possible, hold the contraction for 10 s, and then fully relax the PFM after contraction remaining relaxed for 10 s.
4. One 60-s endurance contraction—the women were instructed to contract the PFM at such a level as to hold it for 60 s.
5. One 60-s rest (post-baseline)—the women were instructed to feel the PFM in resting position.

The following sEMG signal parameters were calculated for our sEMG protocol:

1. One 60-s rest (pre-baseline):
  Average mean amplitude (μV)—the value was calculated separately for each of the 3 intervals: I-5s, II-5s, III-50s.
  Mean amplitude variability (%)—the value was calculated separately for each of the 3 intervals: I-5s, II-5s, III-50s.
2. Five 2-s phasic (flick) contractions with 10-s rest in-between:
  Average peak amplitude from contraction phase (μV)—the result was the mean value from 5 contractions.
  Onset to offset time (s) (contraction duration)—the result was the mean value from 5 contractions.
  Average mean amplitude from rest in-between phase (μV)—the result was the mean value from 5 rests.
  Onset to peak time (s) (time of amplitude increase)—the result was the mean value from 5 contractions.
  Peak to offset time (s) (time of amplitude decrease)—the result was the mean value from 5 contractions.
3. Five 10-s tonic contractions, with a 10-s rest in-between:
  Average mean amplitude (μV)—the result was the mean value from 5 contractions.
  Mean amplitude variability (%)—the result was the mean value from 5 contractions.
  Median frequency (Hz)—the result was the mean value from 5 contractions.
  Mean frequency (Hz)—the result was the mean value from 5 contractions.
4. One 60-s endurance contraction:
  Median frequency (Hz)—the result was the mean value from 6 intervals lasting 10 s each.
  Mean frequency (Hz)—the result was the mean value from 6 intervals lasting 10 s each.
  Average mean amplitude (μV)—the result was the mean value from 6 intervals lasting 10 s each.
  Mean amplitude variability (%)—the result was the mean value from 6 intervals lasting 10 s each.
5. One 60-s rest (post-baseline):
  Average mean amplitude (μV)
  Mean amplitude variability (%)

The sEMG data were filtered using the built-in hardware first-order high-pass filter set to 10 Hz ± 10% cut-off. The raw sEMG data were visually checked for artefacts. The root mean square (RMS) value was determined over a 200-msec window [12,25]. Then, mean and peak amplitude values, and timing parameters were calculated [12,26]. In the Glazer protocol, the signal processing for mean and peak amplitude was performed by averaging sEMG RMS from appropriate phase of this test (average from phase start to end). Average peak amplitude it was mean value of all peaks within evaluated activity. Time before peak and time after peak in the Glazer protocol was calculated strictly according to original procedure implemented in Noraxon MyoResearch software. The resting activity was considered as 0% (minimal) and the peak amplitude was considered as 100% (maximal). The threshold relative at 50% between min and max was considered as the beginning of contraction (rise—before peak) and as the end of contraction (fall—after peak). The signal processing in our protocol was also performed on the basis of sEMG RMS. For resting activity, average mean amplitude was calculated by averaging sEMG RMS within evaluated activity. The average mean amplitude during tonic and phasic contractions (contraction in tonic and rest in phasic) was calculated as a mean value between onset and offset points. The final value was a mean from 5 intervals. The onset time was determined when the signal amplitude reached value above 3 SD of resting mean amplitude. Onset to peak time was calculated as the time between onset and peak (highest amplitude value). Peak to offset time was between peak and offset (point below 3 SD of resting mean amplitude). Fatigue-related changes such as mean and median frequency were calculated using the FFT (fast Fourier transform) in the Glazer protocol and STFT (short-time Fourier transform) in our protocol. The unfiltered RAW sEMG signal was analyzed with a 512-point window over 60 s static contractions. [27,28].

### 2.3. Statistical Analysis

Statistical analysis was carried out using the STATISTICA 12.0 software. To assess the normality of variables distribution, we performed the Shapiro–Wilk test. The between-trial and between-day reliability of the sEMG variables were determined using intraclass correlation coefficients (ICC). We used the ICC (3,1) model according to Shrout and Fleiss [29]. The interpretation of the ICC agreement was performed according to Koo et al. [30]: below 0.50—poor, between 0.50 and 0.75—moderate, between 0.75 and 0.90—good, and above 0.90—excellent. The variability within each set of data was described using coefficients of variation (CV), on the basis of the mean and SD values. Additionally, in order to compare the results of our research with the reliability presented by other authors, we calculated Pearson’s linear correlation coefficient (*r*) for both between-trial and between-day comparisons. The level of statistical significance was set at (*p* < 0.05).

## 3. Results

### 3.1. The Glazer sEMG Protocol

#### 3.1.1. Between-Trial Reliability

The weakest reliability of the Glazer protocol presented muscle timing parameters such as time before peak and time after peak, the median frequency during endurance contraction, and the mean amplitude variability during the pre-baseline resting phase. ICC values for these parameters ranged from 0.05 to 0.37, indicating their poor reliability (Table 1). Moreover, for these parameters, low correlation coefficients (*r*) between the first and second measurements were noted (Table 1). The reliability of other parameters was relatively high, and ICC ranged from 0.68 to 0.95 with a slightly lower value of their correlation coefficient (*r* = 0.52–0.91) (Table 1).

#### 3.1.2. Between-Day Reliability

Analysis of the Glazer protocol reliability between measurements made on different days showed lower values of ICC and correlation coefficients (*r*) compared to their between-trial values. time before peak, time after peak, and the mean amplitude variability during the pre-baseline rest and during endurance contraction phases demonstrated poor reliability (ICC ranged from 0.08 to 0.44 and *r* ranged from 0.08 to 0.39) (Table 2). The reliability of other parameters was moderate, and ICC ranged from 0.54 to 0.75 with the correlation coefficient of *r* = 0.37–0.76 (Table 2).

### 3.2. Our Multi-Activity sEMG Protocol

#### 3.2.1. Between-Trial Reliability

All phases of our protocol demonstrated higher reliability compared to the Glazer protocol. Muscle timing parameters such as onset to offset time, onset to peak time, and peak to offset time showed good and excellent reliability (ICC = 0.85–0.91). The average mean and peak amplitude during rest, flick, tonic, and endurance contractions presented good and excellent reliability (ICC = 0.80–0.95) (Table 3). In our protocol, the reliability of the mean and median frequency during tonic and endurance contractions demonstrated good reliability (ICC = 0.80–0.86) (Table 3).

#### 3.2.2. Between-Day Reliability

The between-day reliability of our protocol was slightly lower compared to between-trial values. Muscle timing parameters such as onset to offset time, onset to peak time, and peak to offset time showed moderate and good reliability (ICC = 0.66–0.90). The average mean and peak amplitude during rest, flick, tonic, and endurance contractions presented moderate and good reliability (ICC = 0.62–0.80) (Table 4). The reliability of the mean and median frequency during tonic and endurance contractions demonstrated moderate reliability (ICC = 0.61–0.75) (Table 4).

## 4. Discussion

The most important observation of this study is that the Glazer protocol commonly used to assess PFM bioelectrical activity is characterized by poor and moderate measurement reliability. The time-domain parameters for the rise and fall of the signal amplitude proposed by Glazer showed poor reliability, which means that timing analysis of the sEMG signal in the original Glazer protocol did not provide information on the temporal characteristics of PFM recruitment. This problem concerns both the between-trial and between-day reliability. Moreover, sEMG signal analysis of muscle fatigue assessment in a 60-s isometric contraction was inconclusive. The poor reliability of the median frequency did not provide useful information regarding fatigue of the tested muscles. Other parameters characterizing the PFM strength (mean amplitude, peak amplitude) indicated mainly good between-trial and moderate between-day reliability. Thus, verification of the measurement reliability of all phases of the original Glazer protocol and all parameters proposed by the authors of this test indicated its moderate diagnostic value.

In this work, the modification of the test performance itself in relation to the Glazer protocol was not large. The greatest changes were made for the registered sEMG signal processing. The main element of our protocol compared to Glazer’s proposal was determination of many additional sEMG signal parameters, which, according to the sEMG signal analysis methodology, allow for assessment of muscle recruitment timing, fatigue, or endurance. In individual phases of the protocol, many additional sub-phases were added, which enabled the calculation of signal parameters not only for the entire phase, but also took signal variability into account during its duration by separate analysis of relevant sub-phases. This approach allowed to largely eliminate errors resulting from incorrect determination of phase boundaries in the protocol and from natural sEMG signal variability.

The results obtained in this way showed moderate to excellent reliability of both time-domain and quantitative parameters of muscle recruitment. The time-domain parameters proposed in our protocol characterizing the muscle recruitment during flick and tonic contractions allowed for reliable assessment of the PFM condition. Moreover, the method of quantitative analysis of the signal amplitude, indicating the amount of muscle recruitment, allowed us to obtain reliable information. In our protocol, the frequency-domain parameters describing muscle fatigue demonstrated much higher reliability than in the Glazer protocol. The sEMG signal processing for each of the six 10-s parts separately and then the averaging the result for them significantly improved the reliability.

Moreover, the diagnostic value of the original Glazer protocol was verified in this study for the first time. The measurement reliability of all phases in the Glazer protocol has not yet been assessed, even by the test author himself. Glazer et al. [18] reported only the Pearson’s correlation coefficient between two measurements, which did not reflect the real reliability of the sEMG signal. Other authors have also reported the reliability of PFM activity, but only during maximal isometric contraction and/or resting state [6,7,8,14,18,19,31].

In this study, for most parameters of the Glazer protocol compared to our protocol, lower reliability coefficients were obtained. Moreover, our protocol demonstrated only slight deterioration of between-day ICC values compared to between-trial ICC. In the Glazer protocol, the between-day ICCs were much lower than the between-trial ICCs. This may have been due to the fact that in the Glazer protocol, the natural variability of the sEMG signal was additionally augmented by the variability resulting from improper signal processing. Within our protocol, a decrease in between-day ICC was also observed, however, only for a few parameters, and this decrease was much smaller than in the Glazer protocol. Perhaps such a result was obtained thanks to the more complex sEMG analysis, taking many specific aspects of signal processing not used in the Glazer protocol into account.

Auchincloss et al. [20] analyzed the reliability of PFM bioelectrical activity using two types of vaginal electrodes. The reliability of between-trial sEMG amplitude regarding short, maximal isometric contractions measured with bipolar electrodes was within the range of ICC = 0.70–0.98, with simultaneous low-sEMG signal variability (CV = 8.5–4.2%). In contrast, between-trial reliability assessed in the reflex contraction when coughing was lower (ICC = 0.58, CV = 20.7%) [20]. Using a unipolar electrode, these authors showed slightly higher between-trial reliability in isometric maximal contraction (ICC = 0.87–0.96, CV = 9.6–16.4%) as well as in reflex contraction during coughing (ICC = 0.81–0.94, CV = 10.6–19.5%). In contrast, the between-day reliability was very low for both maximal isometric contraction and for reflex contraction, respectively, for the bipolar ICC = 0.20–0.57 and for a unipolar electrode ICC = 0.36–0.7 [20]. Moreover, Pearson’s correlation coefficient showed low values *r* = 0.20–0.58 (bipolar) and *r* = 0.37–0.82 (unipolar), respectively, and normalization of the sEMG amplitude did not significantly improve the obtained values [20]. In our study, the peak and mean amplitude value reliability for both flick and tonic contractions concerning the Glazer protocol reached a between-trial ICC, totaling 0.72–0.81, and between-day ICC was equal to 0.51–0.59. However, in the case of our protocol, these parameters demonstrated slightly higher between-trial ICC = 0.73–0.94 and between-day ICC = 0.62–0.80 reliability. In the study by Auchincloss et al. [20], the between-trial reliability was satisfactory, while between-day reliability was low. The authors concluded that the large variability of the between-day results allows this method of PFM assessment to be considered as appropriate when comparing the sEMG signal within a single training session. However, the authors did not recommend comparing the results between measurements carried out on different days. Nonetheless, the author’s research did not confirm these observations, suggesting that sufficiently precise sEMG signal processing allows one to obtain high-quality data that can be used in PFM clinical assessment.

Many works to date in which the reliability of intravaginal PFM bioelectrical activity has been evaluated have had some methodological errors. Thorp et al. [31] examined only eight subjects, and PFM activity was measured only during one short isometric maximal contraction (recorded with a vaginal electrode) and during a 10-s isometric contraction (recorded with an anal electrode). The between-trial reliability of the sEMG signal amplitude was relatively high for both anal and vaginal probes, but simultaneously high variability of the signal amplitude was observed. Moreover, the between-day reliability was expressed only by a correlation coefficient that ranged from *r* = 0.76–0.97, and the authors did not provide a coefficient of variation for the signal amplitude [31].

Thompson et al. [7] observed high reliability of sEMG amplitude in PFMs (ICC = 0.98) measured with a 1-week interval between measurements, but these ICC values were much higher than reported by other authors (ICC = 0.61–0.76) [20]. This was probably due to the fact that Thompson et al. [7] assessed PFM activity using a bipolar electrode, which is more sensitive than a unipolar one. Glazer et al. [18] also reported high reliability of PFM activity using correlation coefficients between two measurements taken on different days (*r* = 0.86, *p* < 0.001). However, the correlation coefficient did not reflect the real measurement reliability because it did not take such factors as signal amplitude variation or measurement error into account. Aukee et al. [8] evaluated the PFM sEMG reliability during short maximal isometric contraction and also reported this measurement reliability as a correlation coefficient. The value was relatively high (*r* = 0.84–0.97, *p* < 0.05), however, the authors underlined that the qualitative visual analysis of the signal collected from the muscles indicated its very large variability [8], and therefore, the reported values seem to be overstated. In our study, the between-day reliability of mean and peak amplitude during tonic contractions in the Glazer protocol was lower (ICC = 0.44–0.59), with the correlation for these parameters being *r* = 0.37–0.42. Moreover, for our protocol, the reliability presented lower values than Glazer et al. [18] and Aukee et al. [8], reaching ICC = 0.73, with a correlation of *r* = 0.58.

The following factor, which may significantly influence the results, is the group homogeneity. In Glazer’s study [18], the group consisted of 37 women within a very large age range: 19–69 years, and included healthy women, females with urinary incontinence, and those with fecal incontinence. This high subject heterogeneity probably had influence on the quality and size of the sEMG signal from the PFM. In our research, appropriate exclusion and inclusion criteria allowed for high subject homogeneity in the cases of age range, absence of any symptoms of urinary incontinence, and non-birth.

Another problem associated with the clinical utility of PFM activity testing is the sequences and type of muscle contractions. There is no doubt that PFM dysfunction is very complex and occurs with disruption of both PFM strength as well as the endurance and coordination of these muscles [4]. Therefore, such complex muscle dysfunction requires equally complex assessment. It seems insufficient to examine the PFM only during maximal contraction lasting from a few to a several dozen seconds. The selective nature of this assessment may lead to false conclusions. In our research, different types of contractions were used in the measurement protocol, and additionally, many parameters characterizing muscle work in various aspects were calculated from each phase of the test.

The limitation of this research is the fact that the study group consisted of young, nulliparous women, aged 20–27 years, without PFM dysfunction. Therefore, due to the better sEMG signal quality measured for non-dysfunctional muscles compared to muscles with dysfunction, the PFM sEMG reliability reported in our study may be higher than in women after childbirth, or in those above the age of 40, in which the bioelectrical activity of PFM may present greater signal variability.

## 5. Conclusions

The most important information obtained in this study was the significant improvement of diagnostic validity in PFM bioelectrical activity evaluation. The high reliability of our sEMG protocol allowed us to suggest that protocol modifications and changes in sEMG signal processing in our methods were effective in the improvement of PFM assessment quality. The new parameters calculated from the sEMG signal which were proposed in our sEMG protocol allowed us to obtain additional clinically important information about PFM dysfunctions, regarding specific deficits of muscle contraction such as decrease in muscle strength; endurance or coordination related to e.g., stress urinary incontinence; or pelvic floor muscle imbalance after childbirth. Moreover, in this study, the reliability of all phases in the original Glazer protocol was evaluated.

### Clinical Perspectives

1. There is a lack of studies in which the reliability of PFM sEMG assessment would be comprehensively evaluated. The purpose of this study was to determine the between-trial and between-day reliability of the multi-activity sEMG measurement protocol for PFM clinical evaluation with the broad aspect of muscle contraction and full signal processing standardisation.
2. The higher reliability of our sEMG protocol compared to original Glazer protocol allowed us to suggest that protocol modifications and changes in sEMG signal processing methods were effective in the improvement of PFM assessment quality. The new parameters calculated from the sEMG signal that were proposed in our sEMG protocol allowed us to obtain additional clinically important information about PFM dysfunctions

## Figures and Tables

**Table 1:** The Glazer surface electromyography (sEMG) protocol between-trial reliability.

Outcome Measure	ICC	*r*	Mean ± SD (1)	CV (%) (1)	Mean ± SD (2)	CV (%) (2)
Rest (pre-baseline)—average mean (μV)	0.83	0.72 *	7.09 ± 3.9	55.6	6.13 ± 3.8	62.0
Rest (pre-baseline)—variability (%)	0.37	0.47 *	10.9 ± 3.5	32.2	10.5 ± 3.1	30.2
Flick contractions—average peak (μV)	0.81	0.69 *	55.2 ± 32.1	58.2	51.4 ± 35.4	68.7
Flick contractions—time before peak (s)	0.05	0.18	0.27 ± 0.10	36.9	0.29 ± 0.1	38.0
Flick contractions—time after peak (s)	0.08	0.11	0.35 ± 0.17	50.4	0.36 ± 0.1	45.8
Tonic contractions—average mean (μV)	0.72	0.59 *	39.6 ± 25.7	64.9	34.9 ± 27.8	79.6
Tonic contractions—average peak (μV)	0.73	0.64 *	47.4 ± 30.5	64.4	42.6 ± 33.6	78.9
Tonic contractions—time before peak (s)	0.12	0.07	1.33 ± 1.35	101.2	1.2 ± 0.9	78.1
Tonic contractions—time after peak (s)	0.70	0.80 *	0.68 ± 0.69	101.4	0.69 ± 0.5	74.8
Endurance contraction—median frequency (Hz)	0.19	0.13	59.0 ± 13.3	22.6	65.0 ± 18.2	92.1
Endurance contraction—average mean (μV)	0.95	0.91 *	17.3 ± 9.06	52.2	16.9 ± 9.6	56.6
Endurance contraction—variability (%)	0.81	0.72 *	17.08 ± 4.5	32.3	20.29 ± 8.9	44.0
Rest (post-baseline)—average mean (μV)	0.95	0.90 *	7.86 ± 4.3	55.0	7.68 ± 4.3	56.5
Rest (post-baseline)—variability (%)	0.68	0.52 *	17.5 ± 14.1	84.0	16.4 ± 12.9	84.3

ICC—intraclass correlation coefficient, *r*—Pearson’s correlation coefficient, SD—standard deviation, CV—coefficient of variation, *—statistical significance (*p* < 0.05).

**Table 2:** The Glazer sEMG protocol between-day reliability.

Outcome Measure	ICC	*r*	Mean ± SD (1)	CV (%) (1)	Mean ± SD (2)	CV (%) (2)
Rest (pre-baseline)—average mean (μV)	0.80	0.68 *	7.09 ± 3.9	55.6	6.19 ± 3.65	59.0
Rest (pre-baseline)—variability (%)	0.39	0.39	10.9 ± 3.5	32.2	10.4 ± 2.9	27.8
Flick contractions—average peak (μV)	0.51	0.41	55.2 ± 32.1	58.2	47.5 ± 27.74	58.3
Flick contractions—time before peak (s)	0.08	0.08	0.27 ± 0.10	36.9	0.28 ± 0.10	36.0
Flick contractions—time after peak (s)	0.10	0.12	0.35 ± 0.17	50.4	0.37 ± 0.17	45.7
Tonic contractions—average mean (μV)	0.54	0.37	39.6 ± 25.7	64.9	33.8 ± 23.9	72.4
Tonic contractions—average peak (μV)	0.59	0.42 *	47.4 ± 30.5	64.4	40.4 ± 29.1	71.9
Tonic contractions—time before peak (s)	0.14	0.11	1.33 ± 1.35	101.2	1.34 ± 1.37	102.2
Tonic contractions—time after peak (s)	0.70	0.76 *	0.68 ± 0.69	101.4	1.02 ± 1.01	98.2
Endurance contraction—median frequency (Hz)	0.16	0.11	59.0 ± 13.3	22.6	62.2 ± 21.0	94.3
Endurance contraction—average mean (μV)	0.76	0.64 *	17.3 ± 9.06	52.2	17.42 ± 11.0	63.1
Endurance contraction—variability (%)	0.44	0.28	17.08 ± 4.5	32.3	18.29 ± 5.7	31.2
Rest (post-baseline)—average mean (μV)	0.77	0.63 *	7.86 ± 4.3	55.0	6.66 ± 3.7	55.0
Rest (post-baseline)—variability (%)	0.68	0.68 *	17.5 ± 14.1	84.0	13.4 ± 7.9	59.0

ICC—intraclass correlation coefficient, *r*—Pearson’s correlation coefficient, SD—standard deviation, CV—coefficient of variation, *—statistical significance (*p* < 0.05).

**Table 3:** Our multi-activity sEMG protocol between-trial reliability.

Outcome Measure	ICC	*r*	Mean ± SD (1)	CV (%) (1)	Mean ± SD (2)	CV (%) (2)
Rest (pre-baseline) phase 1—average mean (μV)	0.83	0.72 *	7.36 ± 3.93	53.4	5.94 ± 3.74	63.0
Rest (pre-baseline) phase 2—average mean (μV)	0.89	0.82 *	7.07 ± 4.01	56.8	5.97 ± 3.73	62.5
Rest (pre-baseline) phase 3—average mean (μV)	0.89	0.81 *	7.06 ± 3.98	56.3	6.16 ± 3.82	61.9
Rest (pre-baseline) phase 1—variability (%)	0.22	0.14	10.29 ± 3.91	38.0	9.80 ± 3.29	33.6
Rest (pre-baseline) phase 2—variability (%)	0.54	0.56	10.38 ± 5.24	50.5	8.78 ± 3.26	37.1
Rest (pre-baseline) phase 3—variability (%)	0.38	0.48	10.55 ± 3.27	31.0	10.19 ± 3.08	30.2
Flick contractions—average peak from contraction (μV)	0.85	0.71 *	57.66 ± 27.3	47.3	47.73 ± 27.8	58.3
Flick contractions—time onset to offset (s)	0.91	0.85 *	0.91 ± 0.38	42.0	0.94 ± 0.41	43.3
Flick contractions—average mean from rest in-between (μV)	0.94	0.81 *	8.20 ± 3.87	47.2	6.71 ± 3.91	58.2
Flick contractions—time onset to peak (s)	0.85	0.77 *	0.36 ± 0.13	37.2	0.38 ± 0.14	37.8
Flick contractions—time peak to offset (s)	0.86	0.77 *	0.51 ± 0.24	48.5	0.52 ± 0.25	47.9
Tonic contractions—average mean (μV)	0.73	0.58 *	30.7 ± 16.4	53.6	29.0 ± 15.0	51.7
Tonic contractions—average mean frequency (Hz)	0.81	0.71 *	88.6 ± 13.8	16.8	90.2 ± 17.7	19.6
Tonic contractions—average median frequency (Hz)	0.86	0.79 *	71.9 ± 12.1	16.8	73.3 ± 16.3	22.2
Tonic contractions—variability (%)	0.83	0.82 *	15.10 ± 3.6	24.0	14.5 ± 3.3	23.1
Endurance contraction—median frequency (Hz)	0.80	0.79 *	62.1 ± 11.7	18.8	62.2 ± 12.8	20.6
Endurance contraction—mean frequency (Hz)	0.82	0.71 *	79.88 ± 13.6	17.1	79.96 ± 12.8	16.1
Endurance contraction—average mean (μV)	0.80	0.67 *	16.34 ± 5.06	49.2	16.9 ± 7.6	47.3
Endurance contraction—variability (%)	0.81	0.84 *	16.10 ± 3.2	28.0	17.5 ± 3.1	26.3
Rest (post-baseline)—average mean (μV)	0.95	0.90 *	7.86 ± 4.3	55.0	7.68 ± 4.3	56.5
Rest (post-baseline)—variability (%)	0.68	0.52 *	17.5 ± 14.1	84.0	16.4 ± 12.9	84.3

ICC—intraclass correlation coefficient, *r*—Pearson’s correlation coefficient, SD—standard deviation, CV—coefficient of variation, *—statistical significance (*p* < 0.05).

**Table 4:** Our multi-activity sEMG protocol between-day reliability.

Outcome Measure	ICC	*r*	Mean ± SD (1)	CV (%) (1)	Mean ± SD (2)	CV (%) (2)
Rest (pre-baseline) phase 1—average mean (μV)	0.78	0.65 *	7.36 ± 3.93	53.4	6.18 ± 3.63	58.7
Rest (pre-baseline) phase 2—average mean (μV)	0.78	0.66 *	7.07 ± 4.01	56.8	6.29 ± 3.74	59.4
Rest (pre-baseline) phase 3—average mean (μV)	0.80	0.68 *	7.06 ± 3.98	56.3	6.18 ± 3.66	59.1
Rest (pre-baseline) phase 1—variability (%)	0.29	0.26	10.29 ± 3.91	38.0	9.07 ± 3.12	34.4
Rest (pre-baseline) phase 2—variability (%)	0.54	0.59	10.38 ± 5.24	50.5	8.42 ± 2.63	31.2
Rest (pre-baseline) phase 3—variability (%)	0.47	0.39	10.55 ± 3.27	31.0	9.99 ± 2.37	23,7
Flick contractions —average peak from contraction (μV)	0.62	0.44 *	57.66 ± 27.3	47.3	43.79 ± 27.5	62.8
Flick contractions—time onset to offset (s)	0.90	0.82 *	0.91 ± 0.38	42.0	0.97 ± 0.45	46.3
Flick contractions—average mean from rest in-between (μV)	0.80	0.50 *	8.20 ± 3.87	47.2	6.04 ± 3.89	64.4
Flick contractions—time onset to peak (s)	0.64	0.52 *	0.36 ± 0.13	37.2	0.33 ± 0.10	30.6
Flick contractions—time peak to offset (s)	0.66	0.68 *	0.51 ± 0.24	48.5	0.59 ± 0.28	48.1
Tonic contractions—average mean (μV)	0.69	0.53 *	30.7 ± 16.4	53.6	27.4 ± 21.9	79.8
Tonic contractions—average mean frequency (Hz)	0.63	0.55 *	88.6 ± 13.8	16.8	84.9 ± 13.9	16.4
Tonic contractions—average median frequency (Hz)	0.75	0.68 *	71.9 ± 12.1	16.8	69.0 ± 17.1	24.8
Tonic contractions—variability (%)	0.56	0.51 *	15.10 ± 3.6	24.0	14.1 ± 3.8	27.1
Endurance contraction—median frequency (Hz)	0.61	0.66 *	62.1 ± 11.7	18.8	57.7 ± 11.7	20.4
Endurance contraction—mean frequency (Hz)	0.73	0.68 *	79.88 ± 13.6	17.1	73.89 ± 11.4	15.4
Endurance contraction—average mean (μV)	0.72	0.75 *	16.34 ± 5.06	49.2	17.12 ± 5.8	51.3
Endurance contraction—variability (%)	0.58	0.48 *	16.10 ± 3.2	28.0	19.4 ± 4.8	33.3
Rest (post-baseline)—average mean (μV)	0.77	0.63 *	7.86 ± 4.3	55.0	6.66 ± 3.7	55.0
Rest (post-baseline)—variability (%)	0.68	0.68 *	17.5 ± 14.1	84.0	13.4 ± 7.9	59.0

ICC—intraclass correlation coefficient, *r*—Pearson’s correlation coefficient, SD—standard deviation, CV—coefficient of variation, *—statistical significance (*p* < 0.05).

## Data Availability

All data generated or analyzed during this study are included in this published article.

## References

[1] DeLancey J.O. (1994). Structural support of the urethra as it relates to stress urinary incontinence: The hammock hypothesis. Am. J. Obstet. Gynecol..

[2] Hodges P.W., Eriksson A.E., Shirley D., Gandevia S.C. (2005). Intraaabdominal pressure increases stiffness of the lumbar spine. J. Biomech..

[3] Lovegrove Jones R.C., Peng Q., Stokes M., Humphrey V.F., Payne C., Constantinou C.E. (2010). Mechanisms of pelvic floor muscle function and the effect on the urethra during a cough. Eur. Urol..

[4] Gunnarsson M., Teleman P., Mattiasson A., Lidfeldt J., Nerbrand C., Samsioe G. (2002). Effects of pelvic floor exercises in middle aged women with a history of naïve urinary incontinence: A population based study. Eur. Urol..

[5] Bø K., Finckenhagen H.B. (2003). Is there any difference in measurement of pelvic floor muscle strength in supine and standing position?. Acta Obstet. Gynecol. Scand..

[6] Thompson J.A., O’Sullivan P.B., Briffa N.K., Neumann P. (2006). Altered muscle activation patterns in symptomatic women during pelvic floor muscle contraction and Valsalva manouevre. Neurourol. Urodyn..

[7] Thompson J.A., O’Sullivan P.B., Briffa N.K., Neumann P. (2006). Differences in muscle activation patterns during pelvic floor muscle contraction and Valsalva maneuver. Neurourol. Urodyn..

[8] Aukee P., Penttinen J., Immonen P., Airaksinen O. (2002). Intravaginal surface EMG probe design test for urinary incontinence patients. Acupunct. Electro-Ther. Res..

[9] Aukee P., Penttinen J., Airaksinen O. (2003). The effect of aging on the electromyographic activity of pelvic floor muscles. A comparative study among stress incontinent patients and asymptomatic women. Maturitas.

[10] Koenig I., Eichelberger P., Leitner M., Moser H., Kuhn A., Taeymans J., Radlinger L. (2020). Pelvic floor muscle activity patterns in women with and without stress urinary incontinence while running. Ann. Phys. Rehabil. Med..

[11] Mense S. (2004). Functional neuroanatomy for pain stimuli. Reception, transmission and processing. Schmerz.

[12] Merletti R., Parker P. (2004). Electromyography: Physiology, Engineering, and Non-Invasive Applications.

[13] Farina D. (2006). Interpretation of the Surface Electromyogram in Dynamic Contractions. Exerc. Sport Sci. Rev..

[14] Navarro Brazález B., Torres Lacomba M., de la Villa P., Sánchez Sánchez B., Prieto Gómez V., Asúnsolo Del Barco Á., McLean L. (2018). The evaluation of pelvic floor muscle strength in women with pelvic floor dysfunction: A reliability and correlation study. Neurourol. Urodyn..

[15] Glazer H.I., Rodke G., Swencionis C., Hertz R., Young A.W. (1995). Treatment of vulvar vestibulitis syndrome with electromyographic biofeedback of pelvic floor musculature. J. Reprod. Med..

[16] Glazer H.I., Hacad C.R. (2012). The Glazer Protocol: Evidence-Based Medicine Pelvic Floor Muscle (PFM) Surface Electromyography (SEMG). Biofeedback.

[17] Hacad C.R., Glazer H.I. (2012). The Glazer Intrapelvic Surface Electromyography (SEMG) Protocol in a Case of Male Urinary Incontinence and a Case of Female Hypoactive Sexual Desire Disorder. Biofeedback.

[18] Glazer H.I., Romanzi L., Polaneczky M. (1999). Pelvic floor muscle surface electromyography. J. Reprod. Med..

[19] Scharschmidt R., Derlien S., Siebert T., Herbsleb M., Stutzig N. (2020). Intraday and interday reliability of pelvic floor muscles electromyography in continent woman. Neurourol. Urodyn..

[20] Auchincloss C.C., McLean L. (2009). The reliability of surface EMG recorded from the pelvic floor muscles. J. Neurosci. Methods.

[21] Delancey J.O., Ashton-Miller J.A. (2004). Pathophysiology of adult urinary incontinence. Gastroenterology.

[22] Zhang Q., Wang L., Zheng W. (2006). Surface electromyography of pelvic floor muscles in stress urinary incontinence. Int. J. Gynaecol. Obstet..

[23] Cornel E.B., van Haarst E.P., Schaarsberg R.W., Geels J. (2005). The effect of biofeedback physical therapy in men with chronic pelvic pain syndrome type III. Eur. Urol..

[24] Oleksy Ł., Mika A., Kielnar R. (2017). Surface Electromiography (sEMG) in the assessment and treatment of pelvic floor muscles. The importance of signal normalization and procedure standardization for interpretation and biofeedback. J. Novel Physiother..

[25] Hermens H.J., Freriks B., Disselhorst-Klug C., Rau G. (2000). Development of recommendations for SEMG sensors and sensor placement procedures. J. Electromyogr. Kinesiol..

[26] Dehail P., Bestaven E., Muller F., Mallet A., Robert B., Bourdel-Marchasson I., Petit J. (2007). Kinematic and electromyographic analysis of rising from a chair during a “Sit-to-walk” task in elderly subjects: Role of strength. Clin. Biomech..

[27] Cifrek M., Medved V., Tonković S., Ostojić S. (2009). Surface EMG based muscle fatigue evaluation in biomechanics. Clin. Biomech..

[28] MacIsaac D., Parker P.A., Scott R.N. (2001). The short-time Fourier transform and muscle fatigue assessment in dynamic contractions. J. Electromyogr. Kinesiol..

[29] Shrout P.E., Fleiss J.L. (1979). Intraclass correlations: Uses in assessing rater reliability. Psychol. Bull..

[30] Koo T.K., Li M.Y. (2016). A Guideline of Selecting and Reporting Intraclass Correlation Coefficients for Reliability Research. J. Chiropr. Med..

[31] Thorp J.M., Bowes W.A., Droegemueller W., Wicker H. (1991). Assessment of perineal floor function: Electromyography with acrylic plug surface electrodes in nulliparous women. Obstet. Gynecol..

